# Coca Erythroxylon

**Published:** 1878-08-20

**Authors:** 


### COCA ERYTHROXYLON.

To a note of inquiry from a medical friend we answer: Little is known, experimentally, of the Coca Erythroxylon in this country, as its introduction is recent. Its effects upon the nervous system are very similar but perhaps more prolonged than those resulting from coffee, producing considerable excitation of the circulatory and nervous systems, and a feeling of exhileration and vigor followed by wakefulness.

In cases of debility from insufficient food as loss of sleep, it tones up the system, increasing the muscular energy and thus proving very useful to soldiers when under long and exhaustive marches. The leaves are not in this market, but the fluid extract may be obtained.

Dr. Verardini, of Bologna, recommends its use in connection with ergot in paralytic cases. A preparation obtained from the leaves, known as the sulphate of cocaina has been found useful in intermittent fevers.

